# Biophysical Sensors Based on Triboelectric Nanogenerators

**DOI:** 10.3390/bios13040423

**Published:** 2023-03-27

**Authors:** Zimeng Ma, Xia Cao, Ning Wang

**Affiliations:** 1 Center for Green Innovation, School of Mathematics and Physics, University of Science and Technology Beijing, Beijing 100083, China; 2 Beijing Institute of Nanoenergy and Nanosystems, Chinese Academy of Sciences, Beijing 100083, China; 3 School of Chemistry and Biological Engineering, University of Science and Technology Beijing, Beijing 100083, China

**Keywords:** triboelectric nanogenerator, biophysical sensor, bioenergy harvesting, self-powered system

## Abstract

Triboelectric nanogenerators (TENGs) can not only collect mechanical energy around or inside the human body and convert it into electricity but also help monitor our body and the world by providing interpretable electrical signals during energy conversion, thus emerging as an innovative medical solution for both daily health monitoring and clinical treatment and bringing great convenience. This review tries to introduce the latest technological progress of TENGs for applications in biophysical sensors, where a TENG functions as a either a sensor or a power source, and in some cases, as both parts of a self-powered sensor system. From this perspective, this review begins from the fundamental working principles and then concisely illustrates the recent progress of TENGs given structural design, surface modification, and materials selection toward output enhancement and medical application flexibility. After this, the medical applications of TENGs in respiratory status, cardiovascular disease, and human rehabilitation are covered in detail, in the form of either textile or implantable parts for pacemakers, nerve stimulators, and nerve prostheses. In addition, the application of TENGs in driving third-party medical treatment systems is introduced. Finally, shortcomings and challenges in TENG-based biophysical sensors are highlighted, aiming to provide deeper insight into TENG-based medical solutions for the development of TENG-based self-powered electronics with higher performance for practical applications.

## 1. Introduction

Since the first invention by Wang et al. in 2012, sensors that are based on triboelectric nanogenerators (TENGs) have attracted strong attention because they can dynamically monitor the physiological condition of our body and environmental stimuli with high sensitivity at the triboelectric interface while converting environmental mechanical energy into electric energy [1]. Due to the advantages of high compatibility, customization, and portability, TENG-based self-powered systems have shown great potential in the ever-growing intelligent device market, and their application is rapidly extending to various fields, including artificial intelligence [2], Internet of things, energy conversion from ocean waves [3], biomedicine [4], disability assistance, and so on, where the TENG is either a power source, a self-powered sensor, or in most cases, both [5].

In principle, materials selection is always the basis for enhancing the performance of TENGs. Currently, many two-dimensional nanomaterials have been proven to be ideal candidates as triboelectric materials, including TMDs, graphene, MXenes, layered MOFs, GO, BP, layered COFs, layered metals, and h-BN [6]. These materials are characterized by ultra-high surface area, adaptability for modification, outstanding electrical conductivity, and optical properties. At the same time, among the many ways to improve the TENG output and sensing performance, 3D fabric structure provides a more efficient, scalable, simple and controllable strategy. The 3D fabric increases the fiber distribution in and out of the plane direction and improves dimensional stability and structural integrity, thereby creating more space for contact separation, which effectively increases the TENG power output density [7]. The 3D fabric structure achieves the asymptotic contact response between fibers and improves the response speed and sensitivity of the TENG [8]. Furthermore, a new type of 4D printed TENG can be designed using spraying technology and fused deposition modelling (FDM) printers. This 4D printing technology is a powerful technique for manufacturing TENGs with self-powered human motion sensors and capturing mechanical energy [9], which is in great demand for accurate robotic sensing and control.

In recent years, with the increasing demand for health, the design and development of TENG-based wearable biosensors in human health monitoring and personalized medicine have garnered significant attention [10]. At the same time, biomechanical energy is one of the most used energy sources. This energy is provided by human joint movement, eyelid movement, respiration, heartbeat, muscle contraction, blood flow, etc. and can be collected by TENGs to generate high-quality electrical signals [11]. As a result, adaptive designs have been intensively developed to integrate TENG-based sensors with the human body in patches, gloves, clothing, and implants to harvest biomechanical energy while fulfilling the wearing requirements of tensile strength and flexibility to provide real-time measurement of physical parameters such as pulse, heart rate, temperature, and blood pressure [12]. With the support of Internet and wireless network technology, sustainable and personalized physiological monitoring of the human body can be carried out, where mobile or portable devices are used to detect, record, and calculate data in vivo to ensure two-way feedback between doctors and patients [13]. In these applications, TENGs can not only quantify various biochemical markers (such as saliva, sweat, skin, and tears) in human body fluids noninvasively, but also be implanted into the human body and provide personalized physiological parameters [14]. At present, TENG-based therapeutic tools, such as a pacemakers, brain nerve stimulators, cardiac defibrillators, and metabolic state monitoring systems, have been developed for clinical treatment [15]. Such innovative designs have shown excellent performance in tissue repair, cell proliferation, and nerve prosthesis [16] with additional advantages in biocompatibility and biodegradability, which also help to reduce the risk of secondary surgery and improve the life quality of patients [17].

**Figure 1:**
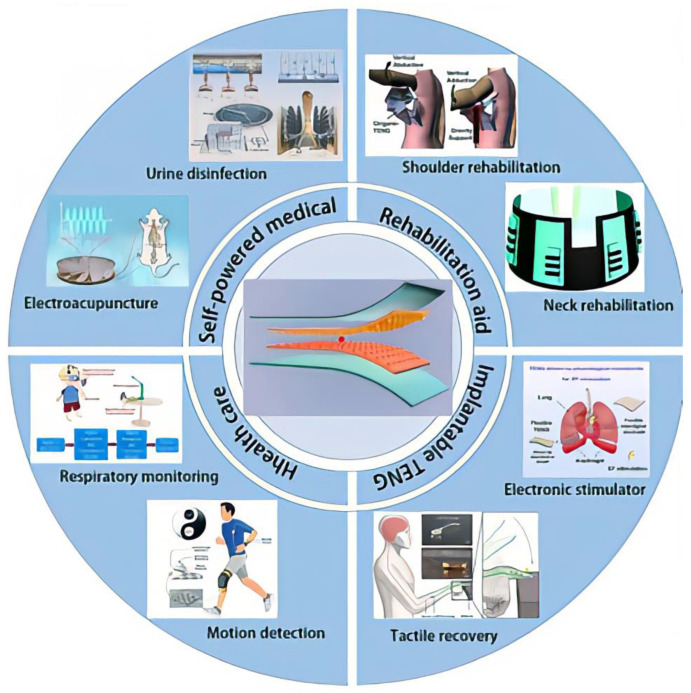
Schematic illustration of TENGs for biosensing, which includes wearable, implantable, and TENG as an external power source for third-party biosensors.

Meanwhile, in the field of biomedicine and healthcare, the power supply of traditional biomedical equipment is limited by a short life span, complex recovery process and large volume [18]. The TENG-based sensor provides the possibility to solve these problems by harvesting the mechanical energy from the motion of internal organs, thus operating in a self-powered mode and reducing the risk of secondary surgery for replacing the power source [19]. With the characteristics of long-term stability, high output performance, wear resistance, and high energy efficiency [20], a TENG-based self-powered system can accurately identify the health status of the body and treat chronic diseases with a flexible plan through continuous feedback of data [21,22]. With innovation and advances in materials science and development in mechanical engineering and wireless communication technologies, the intelligence and mobility of TENG-based self-powered systems have been further optimized for providing customized medical services and personalized medical solutions in drug delivery, wound healing, nerve regulation, respiratory sensing, and so on (Figure 1).

To date, TENG-based biophysical sensors are not yet being marketed, yet new changes in biomedicine are expected, considering the significant potential in health monitoring, prevention, and treatment in biomedicine [23]. Thus, while this review begins from the basic principles and common background of TENGs, the emphasis is put on the current progress in the biophysical sensors that are based on TENGs, including the design criteria, materials selection, and application-oriented consideration in reliability and performance [24]. For clarity, TENG-based wearable sensors are classified into respiratory, cardiovascular monitoring, human rehabilitation, and implantable TENG-based sensors, and their applications in pacemakers, neuroprostheses, and other related areas are discussed separately. Finally, the current shortcomings and challenges of TENG-based biophysical sensors are summarized, and the future development direction and prospects are put forward.

## 2. Working Principles of TENGs

As an innovative energy harvesting device that was initially invented for effectively converting mechanical energy into electrical energy on the base of the electrostatic coupling and triboelectric effect, currently TENGs [25] can be used as both a power source and a sensor for creating self-powered systems for various applications [26]. However, in all the TENG-based integrated systems, TENGs generally work in four modes [27]: vertical contact separation, lateral sliding, freestanding triboelectric layer, and single electrode [28], where advantages of low cost, high efficiency, multiple material selectivity, and miniaturization may enrich various application scenarios [29].

**Figure 2:**
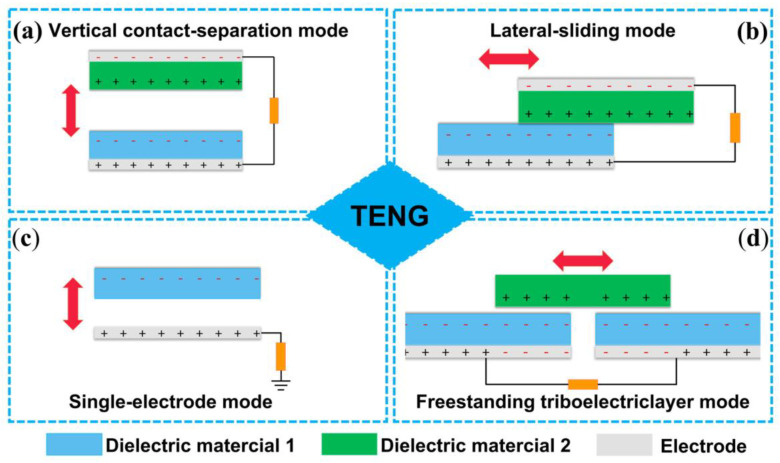
The four fundamental working modes of TENG: (**a**) vertical contact-separation mode [34]; (**b**) lateral-sliding mode [34]; (**c**) single-electrode mode [34]; and (**d**) freestanding triboelectric layer mode [34]; Copyright 2022, Multidisciplinary Digital Publishing Institute.

The vertical contact separation mode: Two dielectric materials with different triboelectric polarities are attached to the back of the current collector respectively [30], and the two triboelectric electrodes are then placed relatively, connected with a closed circuit [31]. When a mechanical force is applied, the contact electrification effect occurs through the vertical contact of the two materials [32]. When the two materials are separated, there is a potential difference between the two materials [33]. Consequently, current is generated in the circuit, and the mechanical energy is converted into electrical energy. In this mode, reducing or increasing the distance between the two materials will change the potential level, and the contact separation cycle is carried out continuously to output alternating current [34]. This mode has advantages such as simple structure, high instantaneous power density, and high working efficiency [35]. At present, many new structures have been developed, such as spring structure, arch structure, and so on (Figure 2a).

Lateral sliding mode: The lateral sliding mode is similar to the vertical contact separation mode [36]. The difference is that the contact mode of the two dielectric materials changes from vertical to horizontal relative sliding, which increases the lateral polarization in the horizontal direction and can output alternating current under periodic sliding [37]. This mode has many forms of existence, such as cylindrical motion, disc rotation. Compared with vertical contact, such designs are more suitable for specific scenarios and have higher energy efficiency (Figure 2b).

Single-level mode: The single-level mode overcomes the design limitations of the two-electrodes design in the above two methods [38]. In this mode, the bottom electrode is grounded, and the electric field distribution is changed by the proximity of the top charged object, so that the charge exchange occurs between the bottom electrode and the earth, resulting in an electric field [39]. The mode has a high degree of freedom, and the motion mode can be in vertical contact or horizontal sliding [40] (Figure 2c).

Freestanding triboelectric layer mode: A pair of symmetrical electrodes are placed under the dielectric layer. There is a gap between the electrodes, which is the same size as the moving dielectric layer [41]. The reciprocating motion of the dielectric layer between the two electrodes drives the electrons to flow back and forth, generating alternating current. This mode does not require a fixed electrode and can obtain mechanical energy from any motions (Figure 2d).

## 3. Current Progress in TENG-Based Biophysical Sensors and Their Medical Application

### 3.1. Current Progress in TENG-Based Biophysical Sensors

For medical applications, TENG-based sensing devices are expected to meet the complex user needs of different people and require a rational tradeoff among high-power output, sensitive response, and satisfactory reliability [42]. The key to solving this problem may lie in the enhancement in biological energy collection efficiency, which may also bring a higher sensitivity of the response to various physiological conditions [43]. Only on the base of high energy conversion efficiency and detection sensitivity, can real-time performance, good stretchability, and high reliability be realized to promote the integration of wearable and implant devices into personalized health services [44]. Consequently, in the journey from discrete TENG to TENG-based sensing systems for biophysical monitoring, structural design, material selection, and surface modification are dominantly important in the pursuit of multi-functionality, high performance, and good stability [45].

#### 3.1.1. Structure Design

Structure design not only increases the consistency of the system, but also greatly reduces the complexity of the integration. Making the device achieve reliable high output stable power supply is the current development trend [46].

**Figure 3:**
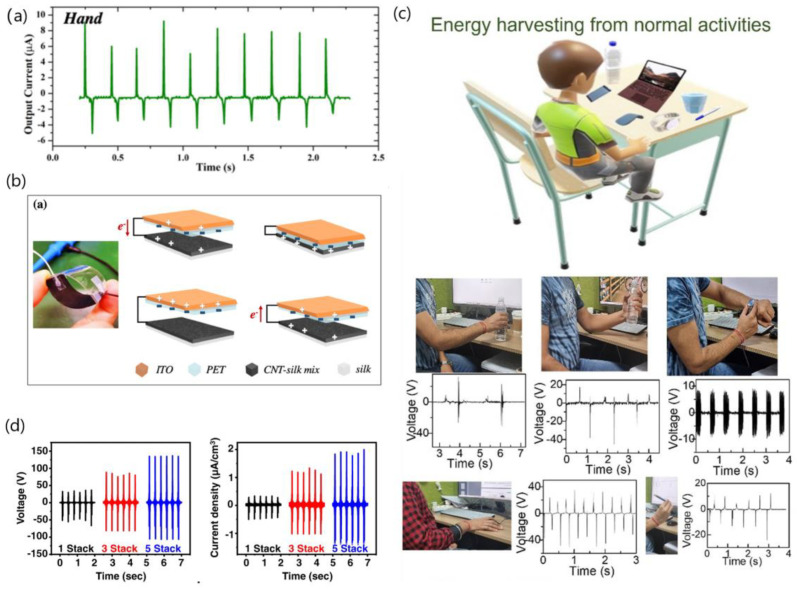
(**a**) The output voltage and current under shaker patting and hand patting [47]; (**b**) The optical image of the arch-shaped testing TENG and the schematic view of the working mode [47]; Copyright 2020, American Chemical Society; (**c**) Diagram of everyday activities from which power could be harvested and images of the corresponding output voltages Copyright 2022, American Chemical Society [48]; (**d**) Voltage output performance and current output performance of one− to five-stacked I-TENGs [49].

Su et al. [47] designed an arching F-TENG with a special layered microstructure (Figure 3a), where the arching design helps to produce an output electricity of 317.4 μw/cm^2^ with hand patting and up to 140.99 μw/cm^2^ with shaker patting (Figure 3b). It can meet the output requirements and produce a high-quality experience when wearing.

Pratap et al. [48] designed a soft and ultra-thin nanostructured TENG. Compared with the non-nanostructured TENG (∼51.6 V), the nanostructured TENG exhibits an open-circuit voltage of ∼128 V when in contact with human skin. In addition, finger touch can produce an output power density of ∼266 μw/cm^2^ (Figure 3c). Such a high performance can be attributed to the higher effective contact area of the latter.

Ryu et al. [49] reported a high-performance commercial coin battery-sized body motion and gravity-based inertial TENG (I-TENG). The output of the as-designed I-TENG can be up to 4.9 μW/cm^3^ in a closed environment and demonstrated excellent performance in recharging lithium-ion batteries (Figure 3d). The device successfully powered implantable medical devices encapsulated in titanium.

#### 3.1.2. Materials Selection

For medical applications, the fabrication of high-quality TENGs is closely related to the selection of reliable and biodegradable/biocompatible materials, whose surface structure, polarity, and microstructure can significantly affect the output performance of devices manufactured from these materials [50]. As a result, how to achieve excellent output performance while ensuring other characteristics such as biological description, stability, flexibility, etc. must be simultaneously considered in the material selection step [51].

Wang et al. [52] used conductive tape and silver fabric as electrodes, mixed with flexible silicone of polyvinylidene fluoride as a dielectric layer, and used polydimethylsiloxane film to package a flexible printed circuit board and the whole TENG. The electrical output performance of the device was improved by nearly three times, and thus a new way for the human movement energy collection was provided.

**Figure 4:**
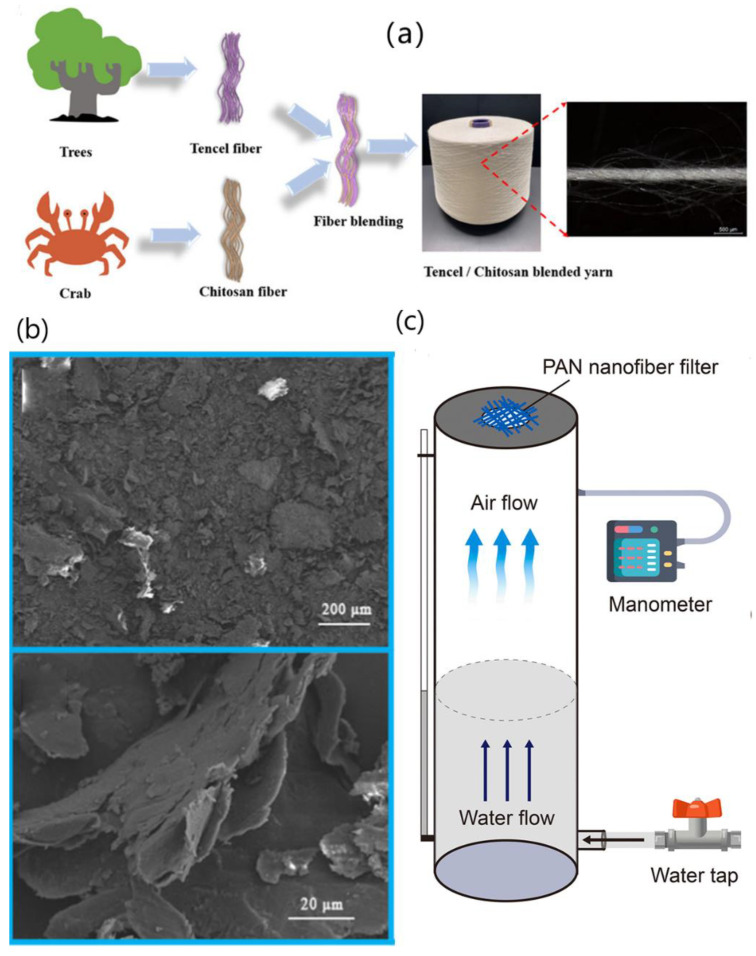
(**a**) Images showing the morphologies of the Tencel/chitosan yarn [53]; Copyright 2021, American Chemical Society. (**b**) SEM images of the surface of the fingernail power in different magnification [54]; Copyright 2020, Elsevier Ltd. (Amsterdam, The Netherlands). (**c**) Schematic illustration of the homemade pressure-drop testing setup [60]; Copyright 2021, Elsevier Ltd.

Xiao et al. [53] designed a scalable, environmentally friendly TENG using chitosan fibers, Tencel, and eco-friendly materials. The output short-circuit current, open-circuit voltage, and power density reached 1.8 μA, 31.3 V and 15.8 mW/m^2^, respectively under the mechanical drive of 3 Hz frequency, 100 N pressure, and the size of 5 × 5 cm^2^ (Figure 4a), which provided impetus for sustainable development.

Fu et al. [54] found that the human fingernail is a high triboelectric material that can be used as the positive electrode material for TENGs to pair with Teflon-based negative electrode material for designing a bio-based TENG. Figure 4b shows the image of the nail force surface under different magnification. This kind of TENG can produce an open-circuit voltage of 87.3 v and a short-circuit current of 3.2 μA at the maximum. With a power density of up to 122 mw/m^2^ generated at both ends of the 23 MΩ resistance, it showed excellent performance in collecting and storing energy from hand movements.

Wu et al. [55] used an implantable rectified Argentum electrode, polydimethylsiloxane, and titanium (PDMS-Ti) in designing a thin film TENG for corrosion protection. The open circuit voltage and short circuit current reached up to 175 V and 14 μA, respectively, which solves the problem of easy corrosion of the implantable magnesium alloy.

#### 3.1.3. Surface Modification

The procedures used in materials selection and surface modification when designing a TENG for other application scenarios are still applicable for the design of TENG-based biophysical sensors [56]. Here the composition and structure of the material can be changed by chemical or physical means, thus endowing the TENG with excellent output performance and other characteristics [57].

Liu et al. [58] designed a self-powered TENG-based artificial joint device to detect wear debris. The device is made by thermo-compression, could in-situ detect wear debris, and has good output performance. It can monitor wear debris in real time, which promotes the development of self-powered artificial joint.

Cheng et al. [59] designed a flexible antibacterial TENG from nanostructured thermoplastic polyurethane (TPU) films. The geometry of nanostructures can be accurately controlled by adjusting the curing conditions of capillary force lithography. Without adding any chemical agent, the device can increase the output power and prevent biofilm formation. Such a design can increase the output power and prevent biofilm formation.

He et al. [60] developed a TENG for respiratory monitoring based on nanofiber-thin film. Due to the unique properties of nanofibers, very small particles can be captured, and the high specific surface area of nanofibers can provide more efficient contact area. By changing the gap distance between the two triboelectric layers to affect the contact area, excellent output performance is obtained (Figure 4c).

### 3.2. Medical Application of Wearable TENG-Based Biophysical Sensing System

TENG-based wearable devices demonstrate many potential medical applications in the healthcare industry [61]. To date, such devices have been promising in improving patient care, tracking personal health data remotely, and even preventing the patient from deterioration or health problems [62]. The high output power of the wearable TENG is still a concern at present. How to maintain the high output while maintaining the stretchability, flexibility, and fit with the skin interface is the key problem in the wearable TENG [63]. As a result, the wearable TENG has promoted the development of new technologies for respiratory monitoring, cardiovascular treatment and detection, and human rehabilitation.

#### 3.2.1. Respiratory Status Monitoring

**Figure 5:**
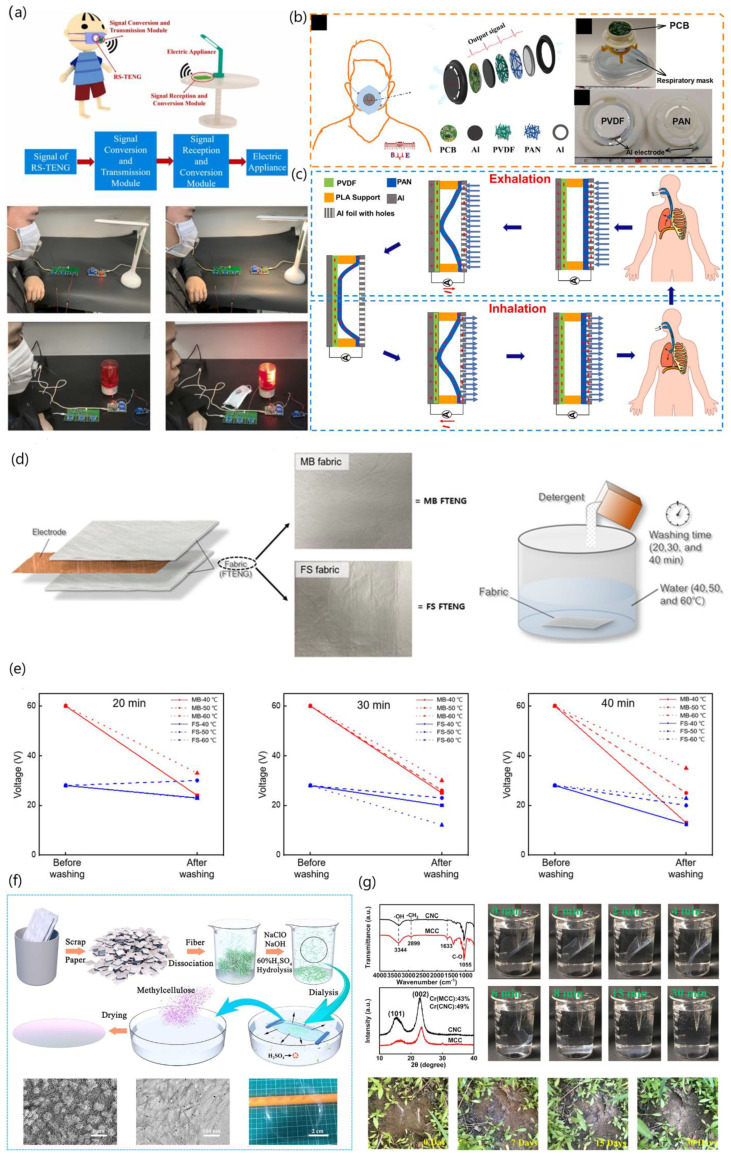
Application of TENG in respiratory monitoring (**a**) The control circuit diagram of the smart facemask; Intelligent Mask driving lamp and alarm system [64]; Copyright 2022, Elsevier Ltd. (**b**) Schematic illustration of the RM-TENG; Image of respiratory mask attached with RM-TENG; The RM-TENG disassembled into two parts [60]; (**c**) Schematic illustration of the working mechanism of the breathing RM-TENG [60]; Copyright 2021, Elsevier Ltd. (**d**) Schematic of a FTENG and photographic surface images of melt-blown nonwoven (MB) and flash-spun nonwoven (FS) fabric sheets and experimental schematic of washing conditions [65]; Copyright 2022, Multidisciplinary Digital Publishing Institute. (**e**) Variation in the maximum peak-to-peak voltage of the FTENGs based on the FS and MB fabrics after washing for 20, 30, and 40 min [65]; Copyright 2022, Multidisciplinary Digital Publishing Institute. (**f**) Fabrication of CNC from wasted paper; SEM image of coarse fiber of wasted paper; TEM image of CNC; Photograph of the CNC/MC film [66]; (**g**) FTIR spectra of CNC and MCC; XRD patterns of CNC and MCC; Photographic images of rapid dissolution of CNC in water and degradation of CNC/MC film in natural environment [66]; Copyright 2022, Elsevier Ltd.

The COVID-19 pandemic urgently calls for the emergence of smart masks to monitor breathing and diagnose physical health. Lu et al. [64] designed a wearable TENG (RS-TENG) for respiration sensing. The RS-TENG can vary with different breathing states, and the respiration output of current and voltage can reach about 0.8 μA and 8 V, respectively. It is proved that by integrating the triboelectric nanogenerator with a mask, the mask has the function of respiration monitoring (Figure 5a). A respiratory alarm system is further built, which combines circuit modules with smart masks to give an alarm when a person stops breathing. Due to its small size, low cost, and simple installation design, the RS-TENG offers a new way to manufacture multifunctional health monitoring tools during the COVID-19 pandemic.

In the clinical monitoring of patients with respiratory diseases, the measurement of parameters such as inhalation time (t_in_), exhalation time (t_ex_), respiratory rate (RR), and their ratio (IER = t_in_/t_ex_) is very important. He et al. [60] developed a respiratory monitoring TENG (RM-TENG) based on nanofiber thin film (Figure 5c). A mathematical model of the influence of the gap distance between two triboelectric layers on the contact area is constructed (Figure 5b). Digital image correlation test (DIC) is used to record the change images of nanofiber layers. Through the analysis of the change curve, it is found that RM-TENG is more sensitive within the range of a small gap between 1 mm and 5 mm. This is because the high specific surface area of nanofibers can bring more effective area contact. After further improvement of RM-TENG parameters and structure construction, the IER and RR values measured by RM-TENG are 93.53% and 100% consistent with the above parameters set by the respirator in real time within 40 h, indicating high monitoring stability. At the same time, the filtration efficiency of 99 wt% can be satisfied in the particle size range of 0.3 μm~5 μm. The RM-TENG can be made into a mask with sensing and filtering features, making it a strong candidate for respiratory monitoring.

RS-TENG can be worn directly to detect the respiratory status anytime and anywhere and can be integrated into the human-computer interface to control the intelligent home through the respiratory status, which is greatly beneficial to the life of the disabled. RM-TENG that is based on nanofiber focuses on higher filtration efficiency so that it can accurately detect a variety of respiratory indicators, with higher accuracy in respiratory monitoring.

Quan et al. [65] studied the TENG (FS FTENG) based on washable flash-spun nonwovens. The PDMS layer inserted into the electrode will increase the output voltage of the TENG nearly five times, which exhibits excellent output performance compared with MB FTENG. By controlling washing conditions, it was found that the fabric structure of FS FTENG after washing was less changed than that of FTENGs (MB FTENG) based on melt-blown nonwoven fabrics used in masks today (Figure 5d). The degradation rate is low and the output voltage attenuation rate is only 12.5%, while the FTENG output voltage attenuation rate based on melt-blown nonwoven fabric is as high as 43.8% (Figure 5e). This result indicates that FS FTENG is more suitable for quarantine mask material.

In the field of human bio-signal monitoring, Wang et al. [66] proposed a water-soluble TENG (WS-TENG) based on water-soluble and biodegradable graphite electrode and recycled paper, respectively. The negative electrode selected ordinary methyl cellulose (MC) film, and the positive electrode selected cellulose nanocrystals (CNC) and MC hybrid CNC/MC film (Figure 5f). For accurate monitoring of various respiratory states, the output voltage range of the WS-TENG was controlled within 0–2 v. The device not only improves output performance but also can be used as a bandage sensor that is very different from the gauze sensor used before, because WS-TENG is water-soluble and can be directly disposed with water (Figure 5g). This water-soluble TENG can be used for real-time monitoring of physiological state signals, which further expands the medical application of the TENG.

#### 3.2.2. Cardiovascular Disease Monitoring and Treatment

Cardiovascular diseases, characterized by high prevalence, disability, and mortality, are common diseases that pose a serious threat to human health [67]. The prevention and monitoring of cardiovascular diseases bring urgent need in innovative medical devices that can provide supplementary information on cardiovascular activities [68].

**Figure 6:**
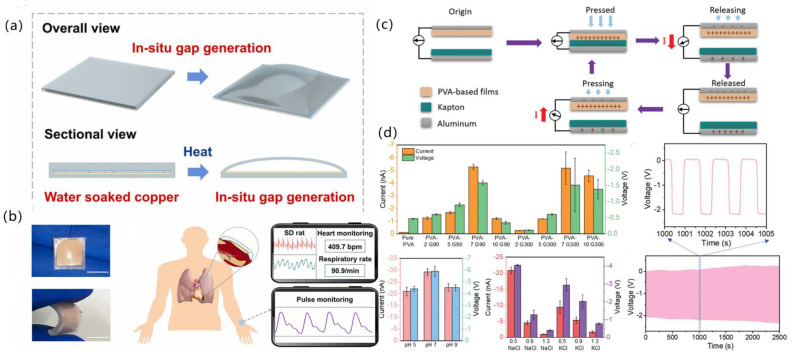
Monitoring cardiovascular information by wearable TENG. (**a**) Schematic diagram briefly showing the in-situ gas gap-generation of the NSTENG [69]; (**b**) Schematic illustration showing the NSTENG to monitor the cardiovascular system [70]; Copyright 2021, Elsevier Ltd. (**c**) Schematic diagram of the working mechanism of PVA-based TENG [71]; (**d**) Summarized currents and voltages outputs of PVA-based TENGs, the stability test of PVA−7G90 sample and the enlarged plot corresponding to the time period indicated as the blue dashed box [71]; Copyright 2020, Wiley-VCH.

Zhao et al. [69] introduced an environmentally friendly method of evaporation soaking distilled water, namely the in-situ air gap-generation method, to prepare a no-spacer TENG (NSTENG) (Figure 6a). This production method not only improves the output performance but also ensures biosafety and avoids air pollution in the vivo. It can withstand larger displacement under the same pressure and achieve a more uniform stress/strain distribution, which can eliminate the small movement caused near the spacer. The application of NSTENG in monitoring the heart movement of rats has achieved a heart rate detection accuracy of 99.73%. The wireless mobile system based on NSTENG can accurately detect the complete pulse waveform and display it in real time on a mobile phone screen (Figure 6b). This method has made great progress in the treatment and monitoring of cardiovascular disease.

Jia et al. [70] fabricated a EPGS-TENG using the doping technique and the in situ gap generation method. The as-designed TENG uses gas as a support layer and Ecoflex-PVDF composite as a negative triboelectric layer. The device has a power output of 121 μW, pressure sensitivity of 7.57 V/N, temperature adaptability of 20° to 40°, and angular response capacity of 374%. The excellent working stability and output performance can simultaneously meet the requirements of self-powered sensing and biomechanical energy acquisition in future biomedicine.

Polyvinyl alcohol (PVA) is one of the polymers widely used in biomedicine. Wang et al. [71] proposed a TENG based on an optimized PVA-gelatin composite membrane for the first time (Figure 6c), the triboelectric properties of PVA blends under the influence of molecular and ionic fillers were systematically characterized and engineered. This TENG not only produces a strong and stable output voltage (Figure 6d) but also provides powerful functionality for detecting subtle skin changes caused by the human pulse without actually capturing the hidden cardiovascular information in the pulse signal. Here the rational design and overall engineering of new materials for more capable biocompatible tribological devices enable continuous monitoring of important physiological signals for self-driven health diagnosis and treatment.

#### 3.2.3. Human Rehabilitation

**Figure 7:**
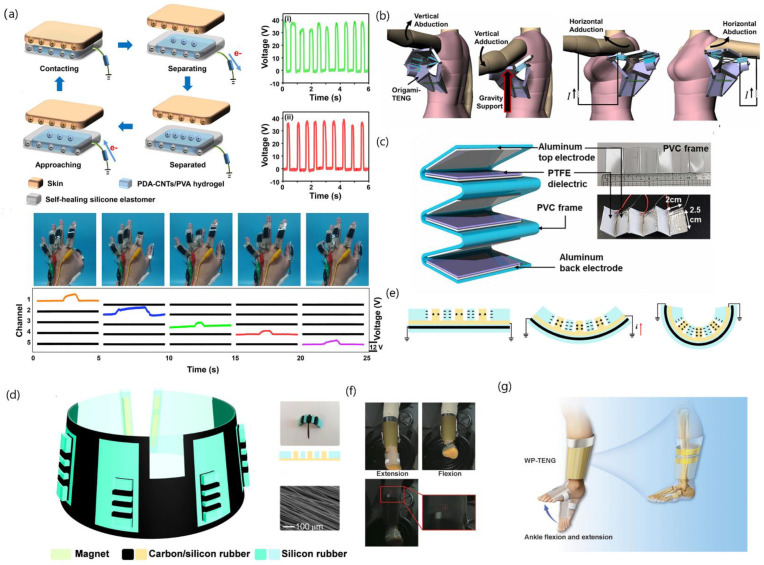
Application of TENG in human rehabilitation. (**a**) Schematic illustration of the working mechanism of the MF-TENG in single-electrode mode; Open-circuit voltage of (**i**) the original and (**ii**) the self-healed MF-TENG; Real-time responses of the bending of fingers acquired via five channels made of the MF-TENG [72]; Copyright 2021, American Chemical Society. (**b**) Schematic showing vertical shoulder adduction-abduction motion with Origami-TENG and Schematic showing horizontal shoulder adduction-abduction motion with Origami-TENG [75]; (**c**) Schematic and photograph of the TENG and its components [75]; Copyright 2022, Elsevier Ltd. (**d**) Schematic structure of the SNM-TS; Photograph of the S-TENG with a carbon fiber line (above) and schematic structure of the S-TENG (below); SEM image of the carbon fiber line [76]; (**e**) Schematics of the operating principle of the S-TENG [76]; Copyright 2022, American Chemical Society. (**f**) WP-TENG is activated by the human limb movement; human ankle flexion and extension drive WP-TENG to form electric flow [78]; (**g**) Schematic illustration of WP-TENG activated by human limb movement [78]; Copyright 2022, Wiley-VCH.

Dan et al. [72] introduced a single-electrode multifunctional TENG (MF-TENG) where the surface of PDA-CNT/PVA hydrogels was coated with self-healing silicone elastomer based on a dynamic imine bond. The PDA-CNT/PVA hydrogel has excellent mechanical properties and can be stretched up to 450%, which makes MF-TENG easy to customize and collect health information at human joints. The short-circuit charge transfer, open-circuit voltage, and short-circuit current of the device are 78.34 nC, 38.57 V, and 7.98 μA, respectively. The device demonstrates not only excellent output performance but also photothermal properties, which is beneficial to human health monitoring. Moreover, even if mechanical damage occurs, it can be restored within 10 min at room temperature. (Figure 7a). It is confirmed that the proposed MF-TENG has a lot of potential in long-term monitoring to help restore one’s health.

Amir Khan et al. [73] developed a kind of tensile, antifreeze and self-healing TENG. The gel network is constructed through the supramolecular interaction of reversible physical bonds, which can achieve rapid self-healing (24 h for the friction layer gel and 4 min for the electrode gel) and provide high tensile properties up to 50 times of strain. The energy collection ability can be exerted in the temperature range of −40° to 80°. The developed gel type TENG can maintain excellent performance even after 5000 cycles, when it is biaxially stretched to 150% strain, its output performance is improved and its elasticity is maintained, gel based TENG has proved to have great potential in energy collection and output.

Jing et al. [74] synthesized a double network hydrogel composed of sodium alginate (SA) and poly (vinyl alcohol) (PVA) and used it as the ion electrode of the hydrogel-based TENG (H-TENG). The elasticity of the hydrogel can be controlled by the concentration of SA. H-TENG can achieve the best tensile property (more than 250%) and high transparency (more than 90%) through the conductivity and viscoelasticity of PVA/SA hydrogel. The peak output current and voltage are 17.6 respectively μA and 203.4 V. H-TENG can easily light 240 blue and green LEDs at the same time. PDMS bags are designed to prevent the dehydration of hydrogel and ensure its stable output. This work reports the characteristics of hydrogel-based TENG.

Patients with impaired shoulder and upper limb function would be greatly assisted in their recovery process if they had access to a gravity support device to maintain their rehabilitation training and exercise function. Divij Bhatia et al. [75] prepared a gravity support device based on integrated TENGs that are used to collect energy and provide power during exercise (Figure 7c). In addition to good output performance, a small enough origami design with a spring is used to construct a gravity support part for rehabilitation exercise under a popular sports games exercise approach (Figure 7b). In the first task, patients required minimal brain-arm coordination effort to play a table tennis game. In the second task, patients gain energy by moving their arms as fast as they can. At the end of the task, patient feedback on the effectiveness of the device was obtained. This work has gained prominence due to how the ongoing pandemic exhibits the potential of products for tele-rehabilitation.

Neck injury is common in all kinds of sports, and the detection of neck motion is an important subject of modern medical treatment. An et al. [76] developed a flexible and energy-saving neck motion detector, which is composed of wearable self-powered TENGs and a deep learning module (Figure 7d). The collar on the detector is integrated by four TENGs based on silicone rubber material. In addition, in order to realize more reliable recognition, a layer of carbon-doped silicone rubber layer should be added between the collar and the sensor to shield the electric field changes caused by the skin. The neck motion state can be judged by the voltage signals of the different output amplitudes and the directions of the four sensors. The combination of these four signals can represent a state of motion. The TENG built from this material not only has high output performance but also has flexibility and tensile properties (Figure 7e). With the help of a convolutional neural network, the deep learning module can identify 11 types of neck movements, including 1 reclining state, 2 twisting directions and 8 bending directions. This design scheme can easily be suited for neck rehabilitation exercises.

It is worth noting that Yao et al. [77] proposed a new functional type TENG based on soft foam material for foot postoperative rehabilitation. Polyvinyl chloride (PVC) tape and soft foam are used as triboelectric materials for this equipment, and the conductive electrode is conductive copper. This kind of TENG improves the output performance and is able to monitor postoperative foot rehabilitation, which can be placed inside shoes to monitor the contact effect of the foot. This research has great application potential in medical rehabilitation.

Bone marrow mesenchymal stem cells (BMSCs) will gradually age with the aging of the human body, and the repair ability and osteogenic potential will also be limited. In medicine, practitioners are faced with the dilemma of how to achieve real-time bone repair in elderly patients. Wang et al. [78] reported a wearable pulse TENG (WP-TENG) (Figure 7g), which uses friction electrical stimulation technology driven by human movement. Bone repair can be achieved with the mechanical sensation of Piezo1 (Figure 7f). Aged BMSCs can optimally enhance osteogenic differentiation, restore vitality, and promote the formation of human umbilical vein endothelial cells (HUVECs) at a peak of 30 μA, which reflects its good output performance. On this basis, WP-TENG using electrical stimulation technology can promote BMSCs osteogenesis and HUVECs angiogenesis has been confirmed. The in vivo study of enhanced bone repair and regeneration further demonstrated that the senescent BMSCs were rejuvenated under triboelectric stimulation of the mechanical channel Piezo1, thereby enhancing the tube formation ability and osteogenic potential of HUVECs.

#### 3.2.4. Application of Textile TENG-Based Biophysical Sensing Systems

In order to effectively collect energy from various mechanical inputs (twisting, pressing, bending, sliding, etc.), the development of TENGs requires a flexible polymer material, especially for the application of human wearable devices. TENGs made from flexible polymer materials have the characteristics of mechanical flexibility, portability, compatibility, and variability [79]. At present, flexible polymers based on textiles or fabrics have developed rapidly in TENG self-powered sensing and energy collection, effectively improving the energy utilization rate and wearing comfort requirements. As the smallest visible unit of fabrics/textiles, fibers are usually prepared by electrospinning, which can ensure high porosity. With horizontal molecular arrangement, controllable thickness, and high surface roughness, more electrospun membranes can provide a variety of lightweight, breathable, and comfortable materials suitable for a skin interface, which is conducive to the production of TENGs based on flexible polymers [80]. Flexible TENGs gradually meet the requirements of antibacterial activity, permeability, washability, waterproofness, adhesion, and comfort in wearable devices, and promote development of flexible TENGs in health monitoring. As a subset of TENG-based wearable electronics [81,82], such TENG-based textile electronics combine the wearing comfort of textiles and the functions of soft TENGs and are in great demand in wearable biophysical sensing applications, so this technology is discussed separately in this review [83].

**Figure 8:**
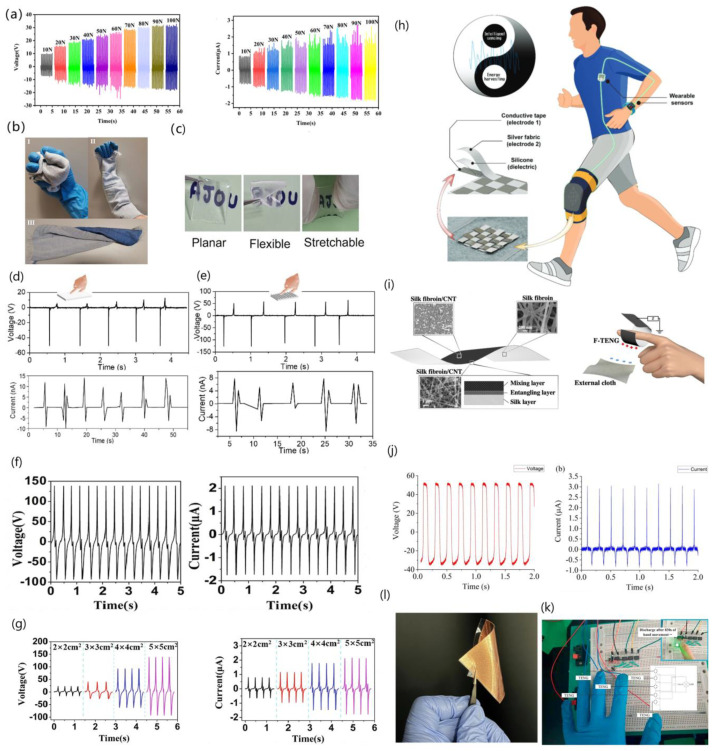
Other applications of wearable TENG. (**a**) Output voltage and current of the FTENG with various impacting forces (the frequency is fixed at 3 Hz and the area is 5 cm × 5 cm) [53]; (**b**) Photographs of the FTENG under different deformations, such as kneaded (**I**), scrolled (**II**), and folded (**III**) [53]; Copyright 2021, American Chemical Society. (**c**) Without the nanostructure and with the nanostructure and short-circuit current [48]; (**d**) Output values of voltage and current [48]; (**e**) The electric output values under different sizes [48]; Copyright 2022, American Chemical Society. (**f**) Application concept of the woven structure TENG. The woven structure can fit closely with the human knee and has many advantages such as good ductility, long service life and low production cost. Silver fabric and conductive tape serve as the electrode, while silicone with a surface pattern structure serves as the dielectric layer. The woven structure kneepad has properties in both energy harvesting and intelligent sensing [83]; (**g**) shows a schematic view of the internal structure of the fabricated F-TENG film and its usage image [83]; Copyright 2021, American Chemical Society. (**h**) The open circuit output voltage of the TENG and the current under corresponding load resistance of 100 KΩ of the TENG [52]; Copyright 2022, Wiley-VCH. (**i**) Photograph of the energy harvesting gloves and its circuit principle and the result of energy harvesting after hand movement for a few minutes [47]; Copyright 2020, American Chemical Society. (**j**) Optical image of PTPU/Cu/PET sandwich TENG structure [54]; (**k**) The resulting TENG is transparent, flexible, and stretchable [54]; Copyright 2020, Elsevier Ltd. (**l**) The structure of single-electrode PTPU-TENG and the right inset is the AFM 3-dimensional images of PTPU films with DVD-pattern (size ∼ 10 μm × 10 μm); Copyright 2023, Elsevier Ltd. [59].

Textile-based TENGs (T-TENGs) have the advantages of being stretchable, cost-effective, and manufacturable. Balancing output performance and flexibility is essential in new wearable electronic devices. Xiao et al. [53] describe a retractable environmentally friendly fabric triboelectric nanogenerator (FTENG). The FTENG with a single electrode mode can output current, voltage, and power density reaching 1.8 μA, 31.3 V and 15.8 mW/m^2^, respectively under the mechanical drive of 3 Hz frequency, 100 N pressure, and the size of 5 × 5 cm^2^ (Figure 8a). The device not only has excellent output performance but also can fit to the skin for comfort, and it has flexibility and excellent antibacterial properties. On this basis, the yarn TENG is manufactured by using the fancy yarn spinning technology, showing the advantages of small diameter, high productivity, high scalability, low weight, and low cost, which provides more possibilities for T-TENG (Figure 8b). The device can be fitted underfoot and to the arm and integrated into multifunctional clothing to detect different body movements. The development of FTENG overcomes the problem that raw materials are often expensive and require tedious chemical processes, and the friction materials used are generally from a non-renewable resource.

The flexible stretchable TENG (FS-TENG) can collect and induce electrical signals during everyday activities, making it an excellent candidate for tactile sensors and energy harvesters. In this work, Pratap Et al. [48] designed a FS-TENG (Figure 8c). Compared with the non-nanostructured TENG (∼51.6 V), the nanostructured TENG exhibits an open-circuit voltage of ∼128 V when in contact with human skin (Figure 8d). This significantly higher value could be attributed to the higher effective contact area of the latter. Using finger touch stimulation as an example, the device can output a power density of ∼266 μw/cm^2^, and it has high signal response and transparency (Figure 8e). With these strengths, the manufactured TENG was used to activate small commercial electronic devices such as light-emitting diodes and implemented as a sensory platform. This work provides more options for TENG to further develop power supplies and wearable sensing.

Zhu et al. [83] designed a fully stretchable textile-based TENG (FSTTN) using the interaction between the electrode@rubber friction and the woven units of the silver-coated glass microsphere (GM@Ag)/rubber electrode (Figure 8f). The as-designed FSTTN (5 cm * 5 cm) can produce 2.1 μA short-circuit current and 138 V open-circuit voltage at 3 Hz flapping frequency, and 0.39 μA short-circuit current and 7.3 V open-circuit voltage at 100% strain (Figure 8g), with excellent output performance. At the same time, due to the excellent tensile properties of the rubber matrix and the high mix ratio of GM@Ag, FSTTN can be conveniently installed on the human body, and various bending movements of the human joints were detected, which provides a new option for motion recognition and medical care.

Wang et al. [52] designed a woven structure TENG. The electrical output performance of the device is nearly 3 times higher due to the design structure of the tip pattern. At the same time, it also improved the comfort of the wearing process. The woven TENG can not only realize self-power supply but also be used to identify waveform changes when human motion is in different states. Taking knee joint bending angle data as an example, machine learning can classify different states of human movement (Figure 8h). The device expands the application of the woven structure TENG for human energy harvesting and sensing.

#### 3.2.5. Other Potential Applications for the Wearable TENG-Based Biophysical Sensing System

Su et al. [47] studied a kind of fiber wikica TENG (F-TENG) based on silk fibroin protein-carbon nanotubes (Figure 8i). The fiber film of this material is converted from a liquid solution. Breaking the traditional thinking of fabricating fiber with metal wire, using the same equipment to run the two steps of electrospray and electrospinning under external conditions, the F-TENG produces an output electricity of 317.4 μw/cm^2^ with hand patting and up to 140.99 μw/cm^2^ with shaker patting. It not only can meet the output requirements but is also durable, simple, and flexible. The device can be fitted to the insole, inner thigh, wrist, or axilla or sewn into a large area of clothing for daily monitoring of the human body. Tests in everyday activities showed that it could charge a capacitor and write by holding a pen or tapping a keyboard, and it could be worn on a finger to drive a humidity thermometer by randomly touching the skin.

Fu et al. [54] designed a bio-based TENG using human fingernails as the positive electrode material and Teflon as the negative electrode material. This kind of TENG can produce an open-circuit voltage of 87.3 V and a short-circuit current of 3.2 μA at the maximum (Figure 8j). With a power density of up to 122 mw/m^2^ generated at both ends of the 23 MΩ resistance, it not only demonstrates excellent output performance but also is cost effective. So, fingernail-based TENGs are designed to collect energy from hand movements (Figure 8k) and store energy in the capacitor to light up the LED. Moreover, it has been designed as a recognition system that can identify different people by relying on pulse signals generated by friction between positive and negative electrode materials. The above work indicates that the human fingernail-based TENG can obtain sustainable renewable electric energy and has great potential in human body feature recognition and energy harvesting.

Cheng et al. [59] developed a TENG that is prepared with nanostructured thermoplastic polyurethane (TPU) film (Figure 8l). The analysis of the correlation between wavelength and amplitude on the output power in this mode can provide a deeper understanding of how a TENG achieves peak output performance. In addition, the device not only improves the output performance but also shows excellent flexibility and antibacterial properties. The device is effective in preventing bacterial growth when attached to the skin. As an innovative application for transparent screen sensors, it can identify specific finger trajectories (for example swipe, hard press, and type) through different electrical responses while providing a solution for bacterial colonization and hygienic problems of users with viral infections when the wearable device is in close contact with human skin.

When implanted in the human body, the high voltage caused by the accumulation of surface charges poses a risk of electrostatic discharge (ESD) in the circuit. There have been satisfactory results to address this problem. A portable ESD prevention system provides a negative charge to the human body through the TENG to keep the human potential stable. After that, the positive charge generated by contact charging of the TENG is compensated by the human movement process, which effectively prevents the ESD effect [84]. Meanwhile, an anisotropic TENG prepared from ordered nanopolymers obtained by electrospinning technology can be rotated 90° when not in operation, thus avoiding charge accumulation and circuit damage [85].

### 3.3. Application of Implantable TENG for Biophysical Sensing System

#### 3.3.1. Cardiac Pacemaker

**Figure 9:**
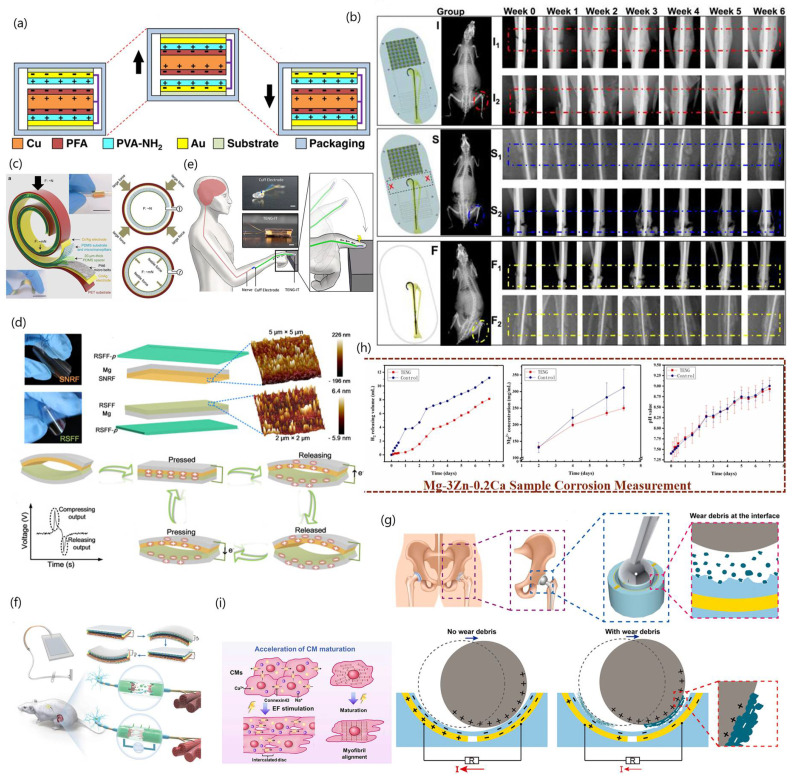
Application of implantable TENG. (**a**) The working mechanism of the I-TENG with the freestanding unit [49]; Copyright 2021, Nature Communications. (**b**) A series of X-ray radiographs on the bone fracture area over time of the intervention group (I), sham group (S), and false-implant group (F) (n = 7) [90]; Copyright 2021, Proceedings of the National Academy of Sciences. (**c**) The device structure of the ATNG; A 20-μm-thick PDMS spacer is used to ensure an ultrashort working distance (20 μm); The inset photos show an actual image of the ATNG [91]; Copyright 2020, Wiley-VCH. (**d**) Appearance of SNRF and RSFF; Schematic illustration of TENG structure; 3D AFM images of friction layers; Schematic diagram of the TENG working principle; Typical output voltage signal during one compression and release cycle [86]; Copyright 2020, Elsevier Ltd. (**e**) Illustration of the TENG-It. a. Use of TENG-IT for restoring tactile sensation [88]; Copyright 2021, American Chemical Society. (**f**) Design and working principle of the self-regulated neural electrical stimulation [87]; Copyright 2022, Wiley-VCH. (**g**) Overview of a TENG-based wear debris detection sensor used in an artificial joint [58]; Copyright 2021, Elsevier Ltd. (**h**) H_2_ releasing volume, Mg^2+^ concentration, and pH value in vitro immersion test of Mg−3Zn−0.2Ca alloy corrosion control through irTENG [55]; Copyright 2022, Elsevier Ltd. (**i**) Electrical stimulation accelerates cardiomyocyte maturation [89]; Copyright 2022, Elsevier Ltd.

Ryu et al. [49] reported an I-TENG with an output power of 4.9 μW/cm^3^, showing excellent performance in recharging lithium-ion batteries (Figure 9a). The I-TENG relies on Bluetooth to monitor the output voltage data in real time and convert mechanical energy into electricity in the body. In addition, the self-charging pacemaker system is integrated with the pacemaker to demonstrate the pacemaker in sensing mode. The device has successfully realized the technological innovation of using biomechanical energy to drive the internal energy collector, which provides the power for the implantable medical device encapsulated in titanium.

Niu et al. [86] developed TENGs based on silk nanoribbon (SNR), which utilized regenerative silk fibroin membrane (RSFF) and nascent SNR membrane (SNRF). RSFF and SNRF have different working capacities and microstructure, and SNRs with a thickness of 0.38 nm are directly exfoliated from natural silk to maintain the original medium/nanostructure (Figure 9d). The device has a maximum current, maximum voltage, and power density of 0.5 μA, 41.64 V, and 86.7 mW/m^2^, respectively. It not only can generate high output energy through human pulse alone but also has high sensitivity. Moreover, the TENG has excellent biodegradability, biocompatibility, and a controllable lifetime composed only of silk and magnesium. Magnesium and silk, as raw materials for TENG, can achieve complete biocompatibility and biodegradation in vitro. Its service life is determined by the post-processing of the RSFF package. This TENG avoids inflammation and second surgery and has excellent performance in output, biodegradability, biocompatibility, and degradation rate, making it very attractive in implant devices and pacemakers.

#### 3.3.2. Neural Prosthesis

One of the most common causes of permanent disability is peripheral nerve damage, and therapeutic electrical stimulation can help regenerate nerves. Some studies have shown that the application of neural prostheses combined with neural cuff implantation has a promising future. However, current electrical stimulation programs cannot complete nerve repair, and there is a lack of research on implantable TENGs. Therefore, Zhou et al. [87] designed an implantable self-regulating sciatic nerve stimulation system, which is composed of contact separation TENGs and nerve cuff electrodes. The injured sciatic nerve can be stimulated by electrical signals generated by the nerve cuff electrodes, and biphasic electrical pulses can be spontaneously generated in response to movement in rats (Figure 9f). With tested biological safety, biocompatibility, and system stability, the output amplitude did not decrease significantly, the skin around the incision was benigngrowth, and the liver and kidney were not deformed. There was no abnormal lymphocyte infiltration and no inflammation, and the body was always in a stable state. Compared with the repair effect of chronic therapeutic electrical stimulation, the upregulation of growth-associated protein may serve as a novel strategy, and the recovery of neurological function can be observed by histological analysis and gait.

Shlomy et al. [88] demonstrated a self-powered integrated tactile TENG. After the device is implanted into the skin, it is transmitted to the sensory neurons through the cuff electrode, which can convert tactile pressure into electric potential, thus stimulating the nerve to simulate tactile sensation (Figure 9e). It not only has good output performance but also is relatively simple, sensitive, flexible, and biocompatible. The demonstration results in rats show that the device provides tactile perception, in which the distal tibial nerve transection blocks the rat’s hindfoot perception. In addition, the tactile pressure exerted on the device determines the degree of electrical activity of induced sensory neurons in vitro. The treatment of tactile loss due to soft tissue injury or traumatic peripheral nerve injury is extremely limited. This work creates a theoretical basis for implantable self-powered TENG in the field of tactile restoration, which is the direction of development prospects.

#### 3.3.3. Cell Maturation

Cardiomyocyte–based therapy is often chosen for the treatment of myocardial injury, but its prognostic ability is limited due to the immaturity of cardiomyocytes. Zhao et al. [89] proposed a TENG-based implantable electrical stimulation device that can be self-powered (Figure 9j). Cardiomyocytes were induced to mature by the electric field generated by the cross-electrode of the device. The cardiomyocytes of newborn rats were significantly promoted to mature in vitro by the addition of c-troponin T, connexin 43, and α-actinin to the device. In addition, the peak Ca^2+^ amplitude in the cardiomyocytes, Ca^2+^ levels, and Ca^2+^ transient rates are significantly improved by electrical stimulation that optimizes fracture formation and sarcomere tissue (Figure 9i). Both the heartbeat of rabbits and the breathing movement of rats can be used as the energy drive of TENG, which is an important technical support for the treatment of myocardial injury.

Guang et al. [90] developed an implantable electrostimulation fracture device consisting of a self-powered TENG with a pair of dressing electrodes that can apply electrostimulations directly toward a fracture. Devices attached to the surfaces of different tissues display biphasic electrical pulses based on surrounding internal movements. The output performance of the device is improved. After electric field optimization, growth factors are activated to regulate the bone microenvironment, promote bone formation and remodeling, and accelerate bone regeneration and maturation. The flexural strength and mineral density increased by 83% and 27%, respectively, compared with the control group, and the fracture healing effect that took more than 10 weeks in the control group was achieved in only 6 weeks (Figure 9b). The high output, battery-free, self-responsive, and bioabsorbable device provides a reliable implantable biological device for the medical treatment of fractures.

#### 3.3.4. Other Implantable Applications

Cheng et al. [91] reported a new type of mechanical asymmetry TENG (ATNG) (Figure 9c), which not only exhibits excellent output performance but also is ultrasensitive. It can even monitor the micro-weak intestinal motion of about 0.3 Hz and can accurately output signals in real time. The physiological states of the gastrointestinal system after glucose absorption at different times have been successfully monitored, despite the presence of multiple noises and disturbances in vivo. This work demonstrates the potential application of TENG for long-term, accurate, and real-time monitoring of weak in vivo microscales.

The injury of articular cartilage often causes long-term pain or even disability and is irreversible. In modern medicine, artificial prostheses are usually used to replace diseased joints for relief. Liu et al. [58] designed a self-powered TENG-based artificial joint device to detect wear debris. The device is made by thermo-compression, consists of polyethylene film and a steel ball, and could in-situ detect wear debris. The simulation test of the fabricated sensor on the worn joint site shows that it not only has good output performance but also can monitor wear debris in real time. In addition, the influence of the size and number of particles on the electronic output signal is also studied. The results show that the voltage decreases gradually with the increase of the number and size of particles (Figure 9g). This work overcomes limitations of the aseptic loosening of wear fragments and promotes the development of TENG in the replacement of diseased joints with artificial prostheses, contributing to the combination of intelligent medicine and biomedical sensors.

Since implantable magnesium alloys are prone to corrosion, Wu et al. [55] designed a TENG (irTENG) in which the open circuit voltage reaches up to 175 V and the short circuit current reaches up to 14 μA. It is confirmed that irTENG can slow down the corrosion rate of Mg-3Zn-0.2Ca alloy in a short-term test (Figure 9h). In addition to excellent output performance, irTENG also has excellent antibacterial activity in experimentation on antibacterial properties of TENG materials. On this basis, the irTENG corrosion protection system was implanted into two-month-developed SD rats. In the long-term implantation experiment on SD rats, it was found that gram-negative and positive bacteria could be effectively eliminated by the Ag electrode. With excellent anti-inflammatory and antibacterial effects in vivo and in vitro, the new bone was obviously better in the process of surgical recovery after implantation, indicating its biocompatibility and potential in vivo application.

### 3.4. Application of TENGs Drives the Third-Party Systems

#### 3.4.1. Cancer Therapy

Cancer chemotherapy exposed patients to low therapeutic efficacy and serious side effects. Qu et al. [92] designed a self-powered drug delivery system using a disk TENG (D-TENG) to provide a current source and a pair of Au electrodes. The current from the D-TENG can stimulate cells within the electrode gap, and the cancer cells can ingest a large amount of the chemotherapy drug doxorubicin when the electrical stimulation is carried out through the self-powered drug delivery system. The peak output voltage, peak output current, and average output current of D-TENG are 135 v, 3.7 μA, and 2.8 μA, respectively at a rotation frequency of about 7.4 Hz, which maintains excellent stability in the long run. The work opens up more possibilities for chemotherapeutic devices.

**Figure 10:**
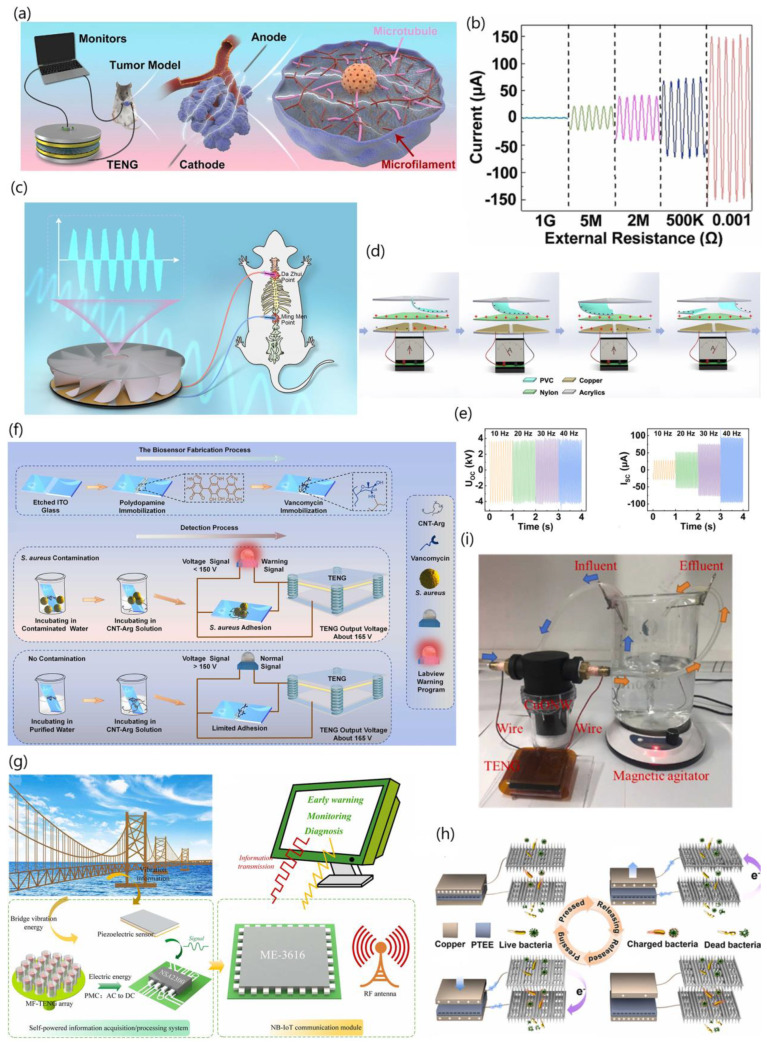
Application of a TENG as self-powered power supply. (**a**) Schematic illustration for the current stimulation of RD-TENG with in vivo system [93]; (**b**) Peak output current with various external resistances at a rotation speed of 120 rpm [93]; Copyright 2022, Elsevier Ltd. (**c**) Concept diagram of EA directly powered by a TENG for functional recovery after stroke, spinal cord injury [94]; Copyright 2022, Elsevier Ltd. (**d**) Working principle and electrical performance of the FS-TENG [95]; (**e**) The open-circuit (OC) voltage and short-circuit (SC) current, respectively, of the FS-TENG with different frequencies from 10 to 40 Hz [95]; Copyright 2022, American Chemical Society. (**f**) Schematic illustration of the TENG driven self-power biosensing system [96]; Copyright 2022, Elsevier Ltd. (**g**) Illustration of the self-powered bridge health monitoring framework and operating mechanism [97]; Copyright 2023, Elsevier Ltd. (**h**) Illustration of the working process of TENG [98]; (**i**) Device picture of T-NEA urine sterilization system [98]; Copyright 2022, Elsevier Ltd.

Metastasis is still a major challenge in cancer therapy. There are still some problems in the reported strategies of metastasis therapy, such as drug side effects, drug resistance, and cell insensitivity to antagonists. Therefore, Chu et al. [93] proposed a therapeutic strategy using electrical stimulation from a TENG (Figure 10a). Taking actin and tubulin related cytoskeleton as an example, they were destroyed under 150μA of TENG output current, thus alleviating cell migration in vitro. Mice inoculated with 4T1-LUC cells were used as tumor metastasis models. The results showed that 150 μA current stimulation effectively prevented the spread of cancer cells to other parts of the body (such as the lung). The results not only exhibited the good output performance of the device but also included no obvious toxicity to other normal organs and tissues (Figure 10b). This electrotherapy alleviates the metastasis of early tumors to lung tissue in vivo, inhibits the cell migration of cancer cells in vitro, and presents a novel strategy with promising potential for cancer metastasis.

#### 3.4.2. TENG-Based Electrotherapy

Electroacupuncture (EA), as a kind of electrical stimulation therapy, is widely used in the biomedical field. Wei et al. [94] inserted the EA into two effective acupoints of rats by applying the dual-phase continuous current generated by a soft-contact suspended rotating TENG (Figure 10c). The results not only demonstrated good output performance but also showed that this method has obvious neuroprotection for spinal nerve contusion in rats. The Basso–Beattie–Bresnahan (BBB) score and gait performance after 2 weeks were improved. The activity of astrocytes at the lesion site was inhibited and the survival of abdominal horn neurons was improved. This suggests that TENG-driven EA therapy may be a medical means to solve traumatic central nervous system injury, creating an experimental basis for TCM treatment.

In medicine, electroporation (EP) is an effective and commonly used method for intracellular delivery. However, the current electroporation method needs a customized high voltage power supply or commercially available EP system. To solve these problems, Liu et al. [95] designed an electroporation system based on a TENG (BEST) (Figure 10d). The device uses a flowing EP unit with capillaries as a resistive load to maximize the load voltage of the TENG and realize impedance matching (Figure 10e). In addition, the experiments involved electrical models, and transfection of BEST indicated that the bulk electric field of the cell medium could reach up to 1 kV/cm, and the transmembrane potential increased by nearly 30 times, thus largely improving transfection efficiency. In addition to excellent output performance, using 40 kDa FITC–dextran indicated that a delivery efficiency above 50% with a cell viability over 90% can be achieved in HeLa cells. This work demonstrates the promising prospect of the TENG as a safe high-voltage power source that provides a low-cost, easy-to-operate solution for EP research.

#### 3.4.3. Other TENG-Driven Systems

Detection of the concentration of gram-positive bacteria is important for assessing water quality and preventing the threat to human health. To achieve the specific detection of gram-positive bacteria, Wang et al. [96] designed a self-powered biosensor system based on vertical contact separation of TENGs, where the signal amplification material is guanidine functionalized multi-walled carbon nanotubes (CNT-Arg) with high electrical conductivity. In this system, the TENG serves as a stable voltage signal source and shows excellent output performance. Due to the specificity of vancomycin-bacterial wall interaction, vancomycin is adopted to identify and capture gram-positive bacterial cells, and the bacterial concentration in solution is detected by measuring the voltage change of the biosensor (Figure 10f). The results indicate that the system has high selectivity and a low detection limit, which promotes the application of TENGs in microbial corrosion, environmental pollution, iatrogenic diseases, etc.

The bridge health monitoring system plays a key role in bridge safety warning, life prediction, fatigue diagnosis, and other aspects. However, the traditional bridge health monitoring system relies on battery or human power supply, which cannot meet the output performance of bridge monitoring sensors. Xia et al. [97] developed a self-powered bridge monitoring system based on an elastic origami structure TENG (EO-TENG) array with a NB-IoTs communication module and a health data acquisition/processing module. The EO-TENG array not only shows good output performance but also provides a stable direct current voltage to a load through an integrated power management circuit (PMC), and the EO-TENG enables the conversion of low-frequency impact and vibration energy into a multi-frequency current output. Vibration signals of piezoelectric components can be obtained and processed by the EO-TENG array with PMC and the drive data processing module of the energy storage device, and then the signals are sent to the terminal by the communication module (Figure 10g). This system provides a new idea for the application of TENGs in bridge monitoring and promotes the development of TENGs in the field of bridge monitoring.

The spread of pathogens will bring negative impacts on ecological environment and human health. Urine is the main source of pathogens, and traditional sewage sterilization has high energy consumption and may produce harmful by-products. Zhang et al. [98] developed a new method for removing bacteria in urine, namely, a TENG-driven nanowire electrode array (T-NEA) system (Figure 10h). Staphylococcus aureus, Pseudomonas aeruginosa, Escherichia coli and Klebsiella pneumoniae cause epidemic infections. This system indicated good output performance, which also demonstrated that the bactericidal efficiency of the artificial urine contaminated by these bacteria was more than 99.9999% through the T-NEA system. A laser confocal scanning microscope and a scanning electron microscope confirmed that this result was due to the irreversible electroporation damage resulting in the inactivation of high efficiency bacteria (Figure 10i). Moreover, T-NEA could effectively degrade the organic components in the artificial urine. This work provides a reliable self-powered driving system solution for the treatment of bacteria in urine.

## 4. Summary and Perspectives

In summary, a brief introduction to the development of various kinds of TENG-based sensors and self-powered systems for biophysical sensing is provided in this review. While covering various innovative strategies from the perspective of structural design, materials selection, and surface modification, the balance among the output performance, reliability, and sensitivity of TENG-based biophysical sensors and self-powered systems is emphasized for various application scenarios, including in respiratory and cardiovascular monitoring and human body parts recovery. The latest achievements of the implantable self-powered TENG in myocardial injury, touch recovery, cardiac pacing, corrosion protection, micro-monitoring, and bone therapy are also discussed. The advances of the self-powered device integrated with other electronic devices in sterilization, cancer therapy, and health monitoring are succinctly summarized.

However, to bridge the span from laboratory scale to practical application, future research on TENG-based biophysical sensing systems can be strengthened in the following aspects.

### 4.1. Improvement in Durability and Reliability

Most TENG-based biophysical sensors are in contact with our skin for a long time, and the performance degradation caused by high-frequency wear cannot be avoided during operation. In terms of material selection, traditional textile materials, such as nylon, silk, and cotton, have attracted much attention recently. Compared with other materials that have been utilized previously, such as silicone polymer, the use of these materials in triboelectric layers significantly improves the durability of the device with the design of switchable structures to convert the TENG’s contact into non-contact modes.

### 4.2. Enhancement in Output Performance

The output performance is crucial for the TENG to meet the requirement of practical applications. From the internal structure, contact can be enhanced, surface charge density can be improved, and new TENG structures can be developed by adjusting the surface structure of the friction layer and modifying triboelectric active materials. From the external structure, power management circuits can be selected, integrated systems can be created, and environmental conditions can be optimized to improve the output performance. Further improvement in the lubrication fluid may also help to reduce the friction coefficient and improve the energy conversion efficiency.

### 4.3. Durability

TENG is made of polymer materials, and most of the research is driven by electric motors. Under the influence of a harsh environment and the high-frequency friction of body movement, TENG wear will be caused and its service life will be affected. There is an urgent need to develop wear-resistant TENGs with high performance. In recent years, self-healing materials, which have anti-twist, sliding, and mechanical bending characteristics, are expected to play an important role in durability.

### 4.4. Stability

In order to reduce the replacement and maintenance of implantable TENGs, in the real-time detection of artificial prosthesis wear, the TENG should overcome the limitation of debris loosening for enhanced stability. In the future, we can focus on the development of an energy supply system based on energy conversion in biological body, which can effectively extend the battery life and greatly correct the power supply defects. Self-healing and biocompatible materials should be explored to achieve a stable and sustainable power supply.

### 4.5. Degradability

At present, most of the synthetic polymers used for the design of TENGs are non-degradable, which severely limits the application of TENGs in humans and poses a threat to the ecological environment. It seems wise to choose degradable materials. However, while TENGs based on silk nanoribbon exhibit excellent biodegradability and the degradation rate reaches a satisfactory level, such materials often do not meet the expectation of mechanical properties. In the future, new physical or chemical methods should be explored to improve these properties and more biodegradable natural materials should be found, which will greatly promote the development of TENGs.

### 4.6. Intelligence

With the development of the Internet of Things and 5G, big data analysis based on TENGs can help to expand the application range of TENGs. In the development of smart masks, for instance, the breathing state is successfully used to control the smart home, which greatly increases life quality. By combining with artificial intelligence to change the methods of medical treatment, the data transmission, analysis, and feedback via wireless networks can support the freedom and convenience of medical applications and should be the focus of future research for the development of wireless sensors in the network era.

## Data Availability

Data available on request from the authors.
